# Nanocomposite Scintillators Loaded With Hafnium Oxide and Phosphorescent Host and Guest for Gamma Spectroscopy

**DOI:** 10.1021/acs.chemmater.4c00805

**Published:** 2024-05-07

**Authors:** Isabelle Winardi, Ziqing Han, Hao Yu, Prabhav Surabhi, Qibing Pei

**Affiliations:** Department of Materials Science and Engineering, California NanoSystems Institute, University of California, Los Angeles, Los Angeles, California 90095, United States

## Abstract

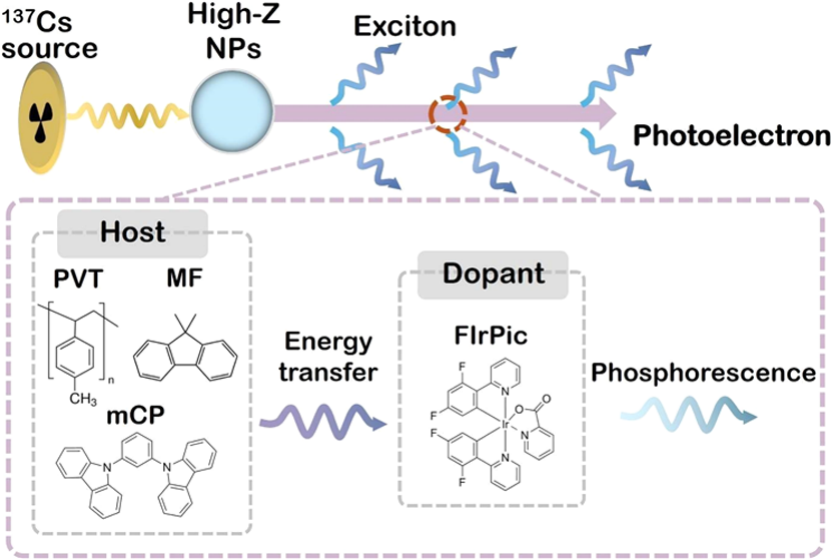

Gamma sensitive plastic scintillators are of critical importance in the fields of nuclear nonproliferation, medical imaging, and high energy physics. However, there is often a trade-off between high light yield and high loading of high-*Z* components, both of which play an essential role in gamma ray detection. This work takes advantage of triplet exciton harvesting to increase gamma light yield by utilizing 1,3-di(9*H*-carbazol-9-yl)benzene and 9,9-dimethyl-9*H*-fluorene as triplet hosts to facilitate Dexter energy transfer to bis[2-(4,6-difluorophenyl)pyridinato-C2,N](picolinato)iridium(III) (FIrPic), a blue light emitting phosphorescent dye. A plastic scintillator containing 20 wt % MF, 10 wt % mCP, and 2 wt % FIrPic has a high gamma light yield of 14 800 Ph/MeV. Incorporating 20–35 wt % hafnium oxide nanoparticles into this organic matrix results in nanocomposites that demonstrate a gamma photopeak energy resolution of 6.4–9.7% at 662 keV while still retaining a high gamma light yield between 8800 and 10 800 Ph/MeV.

## Introduction

Plastic scintillators are popular for radiation detection applications due to their low cost, environmental stability, and ease of fabrication, operation, and maintenance, which explains their widespread use in the fields of nuclear nonproliferation, medical imaging, and high energy physics.^1,2^ For applications involving radioisotope identification, large scale scintillators are required for practical counting rates. Large scintillators on the order of several cubic meters are especially necessary for radiation portal monitors customarily used for scanning radioactive nuclear agents.^3^ Compared to inorganic scintillators, however, the light yield of plastic scintillators is relatively poor. Besides, unloaded plastic scintillators, due to their low density and low atomic number (*Z*), are unsuitable for applications that require high energy resolution or for gamma ray detection.^4^ Upon first glance, plastic scintillators may appear to have many disadvantages, but overall, they are actually more promising than many other detection technologies. For example, gas ionization and semiconductor detectors, while they offer high performance, are often expensive and difficult to operate and maintain. Moreover, crystalline and ceramic scintillators, which boast high light yields, are high cost and are not environmentally stable due to their hygroscopic nature.^5^

Here is where high-*Z* plastic scintillators enter the picture, combining the low cost, fast response, and scalability of traditional plastic scintillators with the high-*Z* elements necessary for high resolution nuclear monitoring. Using high-*Z* plastic scintillators for spectroscopic identification of radionuclides is made possible by comparing the measured energies that result from the interaction of gamma rays with high-*Z* elements against known radionuclide standards.^6^ Thus, as long as plastic materials demonstrate satisfactory light yield and are able to incorporate high-*Z* elements, they remain viable competitors against other gamma radiation detection technologies, as shown by our group’s previous work that incorporated high-*Z* nanoparticles into a fluorene-enhanced organic matrix.^7,8^ Unfortunately, these high-*Z* plastic scintillators often see a trade-off between high light yield and high loading of high-*Z* components, as high-*Z* nanoparticles are non luminescent and photoelectric energy deposited in the nanoparticles do not contribute to the scintillation light yield. Therefore, the development of a plastic matrix with exceptionally high light yield becomes critical, especially at high nanoparticle loading.

High light yield entails efficient energy transfer from the high energy excitons generated during the deposition of photoelectric energy to the lower band gap luminescent dyes, followed by outcoupling of the resulting low energy photons.^9^ Ordinarily, ionizing radiation produces both singlet excitons and triplet excitons. In conventional plastic scintillators, only the singlet excitons are converted into emitted photons through fluorescent energy transfer, meaning that the triplet excitons, although they still transition from the excited state to the ground state, do so in a nonradiative manner that does not result in any photon emission.^10,11^ By utilizing phosphorescent dyes that take advantage of triplet excitons, the light yield may be increased.

**Figure 1:**
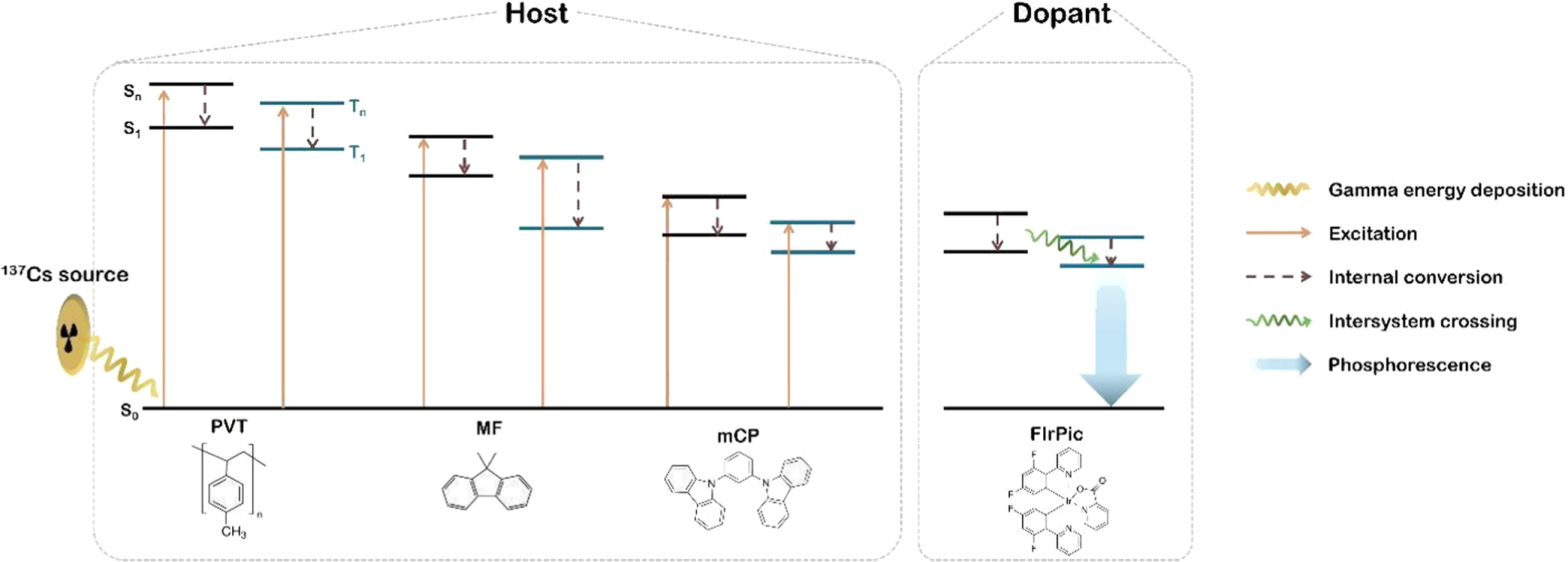
Energy levels responsible for FRET and Dexter energy transfer between host and dopant.

Phosphorescent molecules are already widespread in organic light emitting diodes (OLEDs), with some devices approaching an internal quantum efficiency of 100%, highlighting the efficacy of utilizing both singlet excitons and triplet excitons.^12,13^ One particular system uses 1,3-di(9*H*-carbazol-9-yl)benzene (mCP) as the host and bis[2-(4,6-difluorophenyl)pyridinato-C2,*N*](picolinato)iridium(III) (FIrPic) as the blue light emitting dopant.^14−16^Figure 1 illustrates the energy levels in a scintillation system comprising mCP and FIrPic, as well as polyvinyltoluene (PVT) as the plastic matrix and 9,9-dimethyl-9*H*-fluorene (MF) as an intermediate cascade energy transfer agent. Förster resonance energy transfer (FRET) is effective over a distance of up to 10 nm, whereas Dexter energy transfer operates up to a distance of 1 nm.^17^ Efficient FRET occurs between PVT and MF as well as between PVT and mCP, whereas Dexter energy transfer is the mechanism responsible for the energy transfer between MF and FIrPic as well as between mCP and FIrPic.^18^

Polyvinylcarbazole scintillators employing FIrPic as the fluor and 40 wt % triphenyl bismuth as the high-*Z* material have been reported, producing high light yields of over 30 000 Ph/MeV, measured for β radiation. When looking at the gamma light yield, however, the same scintillator has a light yield of just over 7000 Ph/MeV.^19−21^ Besides, the vinylcarbazole monomer has a melting point of 65 °C, making it an unsuitable candidate for dissolving nanoparticles at room temperature, explaining the necessity of using PVT for our nanocomposites. In this work, we demonstrate a phosphorescent nanocomposite loaded with 35 wt % hafnium oxide nanoparticles. Through the incorporation of both MF and mCP as the host materials and FIrPic as the dopant, the resulting nanocomposite retains a gamma light yield greater than 8800 Ph/MeV.

## Experimental Section

### Materials

1,3-Di(9*H*-carbazol-9-yl)benzene (mCP, 98%) was purchased from Aaron Chemicals. Hafnium(IV) chloride (99%) was purchased from Acros Organics. Bis[2-(4,6-difluorophenyl)pyridinato-C2,*N*](picolinato)iridium(III) (FIrPic) was purchased from American Dye Source. Ethanol was purchased from Decon Laboratories. EJ-550 optical grade silicone grease was purchased from Eljen. Trifluoroacetic acid was purchased from EMD Millipore Corporation. Acetone (Certified ACS), hydrogen peroxide (Certified ACS, 30%), sulfuric acid (Certified ACS Plus), and toluene (Certified ACS) were purchased from Fisher Chemical. Bis(2-(methacryloyloxy)ethyl) phosphate, 1,1-bis(*tert*-butylperoxy)-3,3,5-trimethylcyclohexane (Luperox-231, 92%), cyclohexane (99.9%), divinylbenzene (technical grade, 80%), inhibitor removers (Al_2_O_3_), methylstyrene (60% *meta*, 40% *para*, and 1% *ortho*, 99%), and oleylamine (technical grade, 70%) were purchased from Sigma-Aldrich. 9,9-Dimethyl-9*H*-fluorene (MF, 97%) was purchased from Synthonix. Chloroform (99.8%) was purchased from Thermo Scientific. Trichloro(1*H*,1*H*,2*H*,2*H*-tridecafluoro-*n*-octyl)silane was purchased from Tokyo Chemical Industry. Methylstyrene and divinylbenzene were purified by a column packed with inhibitor removers to remove *tert*-butylcatechol, then degassed before use. Luperox-231 was also degassed before use. All other materials were used as received.

### Synthesis of Hafnium Oxide Nanoparticles

Hafnium oxide nanoparticles were synthesized according to a literature protocol.^8,22,23^ Typically, 50 mL trifluoroacetic acid was added dropwise to 30 mmol (9.6 g) hafnium(IV) chloride, then stirred at 40 °C overnight. Purification was performed through rotary evaporation and vacuum drying, yielding hafnium trifluoroacetate. Then, 10 mmol (6.3 g) hafnium trifluoroacetate and 100 mL oleylamine were loaded into a round-bottom flask. After degassing the reaction mixture, the flask was heated to 340 °C under an argon atmosphere. The reaction was run for 30 min, then cooled to room temperature. Purification of the nanoparticles was performed through flocculation with ethanol and acetone. The purified nanoparticles were dissolved in toluene and saved for further use.

### Synthesis of Plastic Scintillators and Nanocomposites

For surface modification of the hafnium oxide nanoparticles, the pristine oleylamine ligand was partially exchanged by bis(2-(methacryloyloxy)ethyl) phosphate as described in a literature protocol.^8,22−27^ After ligand exchange, the nanoparticles were vacuum-dried, transferred into a nitrogen-filled glovebox, and dispersed into a degassed formulation containing 5 vol % divinylbenzene in methylstyrene, after which FIrPic, Luperox-231 (thermal initiator, 0.1 vol %), mCP, and MF were dissolved into the solution. The curing solution was transferred to a glass vial (cleaned with 3:1 sulfuric acid/30 wt % hydrogen peroxide and treated with trichloro(1*H*,1*H*,2*H*,2*H*-tridecafluoro-*n*-octyl)silane) and sealed with a plastic lid and polytetrafluoroethylene (PTFE) film. The solution was cured at 95 °C for 18 h, yielding a transparent monolith that was demolded, grinded, and polished before characterization. All monoliths contained 2 wt % FIrPic. Meanwhile, nanoparticle loading varied from 0 to 35 wt %, mCP loading varied from 0 to 10 wt %, and MF loading varied from 0 to 20 wt %.

### Characterization

Transmission electron microscopy (TEM) was performed using an FEI Tecnai T12 TEM. The nanocomposite was etched by a focused ion beam using a Nova 600 SEM/FIB System to expose a cross section and transfer it to a TEM grid for imaging. Thermogravimetric analysis (TGA) was performed using a PerkinElmer TGA 8000 by heating the samples to 850 °C under an air atmosphere. UV–vis data were obtained from a Shimadzu UV-1700 spectrophotometer. Solution samples in cyclohexane were loaded into a borosilicate glass cuvette with a light path of 1 cm, while plastic scintillator and nanocomposite samples were adhered to a glass slide with EJ-550 optical grade silicone grease. Photoluminescence data of solution samples in cyclohexane were obtained from a PTI QuantaMaster 30 spectrofluorometer.

### Gamma Scintillation Measurements

Measurements were performed using a home-built system constructed in a light proof box and follow a previously reported literature protocol, with minor modifications.^7,8,26^ Typically, the plastic scintillator or nanocomposite was placed in a PTFE sample holder and coupled to a Hamamatsu R878 photomultiplier tube (PMT) using EJ-550 optical grade silicone grease. A Cs-137 source was placed outside the PTFE sample holder, and a PTFE back reflector was used to optimize photon collection by the PMT while also excluding the influence of concomitantly emitted β rays from the Cs-137 source. The PMT was equilibrated for 30 min after sealing the light proof box with high performance black masking tape. Typical acquisition live time varied between 1 and 9 h. The signal was recorded by a Canberra Lynx multichannel analyzer with rise time and flat top time set to 2.2 and 3.2 μs, respectively. Scintillator gamma light yield was obtained by comparing the sample’s Compton edge channel number to that of a standard EJ-212 sample tested under the same conditions, followed by correction with regard to the sample’s transmission mode photoluminescence spectra. Transmission mode photoluminescence data were obtained from a home-built system using an excitation wavelength of 365 nm.

## Results and Discussion

### Synthesis and Characterization

**Figure 2:**
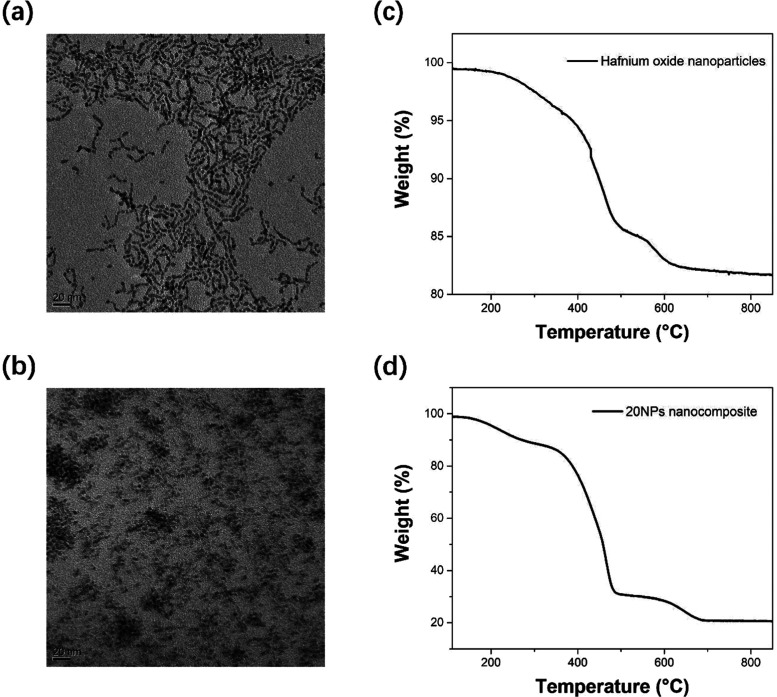
Characterization of nanoparticles and nanocomposites. (a) TEM image of hafnium oxide nanoparticles with oleylamine ligand. (b) TEM image of a focused ion beam etched thin film from nanocomposite containing 20 wt % nanoparticles. (c) TGA curve of hafnium oxide nanoparticles (NPs) taken under air atmosphere. (d) TGA curve of nanocomposite containing 20 wt % NPs.

Hafnium oxide nanoparticles were synthesized according to a literature protocol at the scale of 2–3 g product per batch for an inorganic yield of approximately 95%.^8,22,23^ Typically, hafnium trifluoroacetate and oleylamine were heated at 340 °C for 30 min under an argon atmosphere. Purification of the nanoparticles was performed through flocculation with ethanol and acetone. The transmission electron microscope (TEM) image in Figure 2a illustrates that the nanoparticles have a uniform size distribution and are approximately 4 nm in diameter. Before incorporating these nanoparticles into the nanocomposites, surface modification was performed by partially exchanging the oleylamine ligand with bis(2-(methacryloyloxy)ethyl) phosphate, allowing subsequent copolymerization of the phosphate ligand with methylstyrene during curing.^8,22−27^ After ligand exchange, the nanoparticles were dried, transferred into a nitrogen-filled glovebox, and dispersed into a formulation containing bis[2-(4,6-difluorophenyl)pyridinato-C2,N](picolinato)iridium(III) (FIrPic), 1,1-bis(*tert*-butylperoxy)-3,3,5-trimethylcyclohexane (Luperox-231), 9,9-dimethyl-9*H*-fluorene (MF), 1,3-di(9*H*-carbazol-9-yl)benzene (mCP), divinylbenzene, and methylstyrene. The curing solution was transferred to a glass vial, sealed, and cured at 95 °C for 18 h, yielding a transparent monolith that was demolded, grinded, and polished before characterization. All plastic scintillators and nanocomposites contained 2 wt % FIrPic. Meanwhile, nanoparticle loading varied from 0 to 35 wt %, mCP loading varied from 0 to 10 wt %, and MF loading varied from 0 to 20 wt % in the plastic scintillators and nanocomposites. There is a uniform dispersion of nanoparticles within the nanocomposite, as indicated by the TEM image in Figure 2b showing a focused ion beam etched thin film from a nanocomposite containing 20 wt % nanoparticles.

The TGA curve of the hafnium oxide nanoparticles performed under an air atmosphere is shown in Figure 2c and illustrates a normalized residue weight of 82% at 850 °C, corresponding to an oleylamine ligand weight percentage of 18 wt %. TGA was also performed on a nanocomposite containing 20 wt % nanoparticles, resulting in a normalized residue weight of 21% at 850 °C as shown in Figure 2d. This residual weight represents the actual hafnium oxide nanoparticle loading excluding the organic ligands. Accounting for the weight of the ligands results in an overall loading of nanoparticles containing surface ligands that is approximately 25%, which actually exceeds the 20 wt % loading in the nanocomposite formulation. This positive deviation persists with nanocomposites containing higher nanoparticle loadings, and is likely caused by the evaporation of a small fraction of methylstyrene during curing at 95 °C.

### Optical Properties

**Figure 3:**
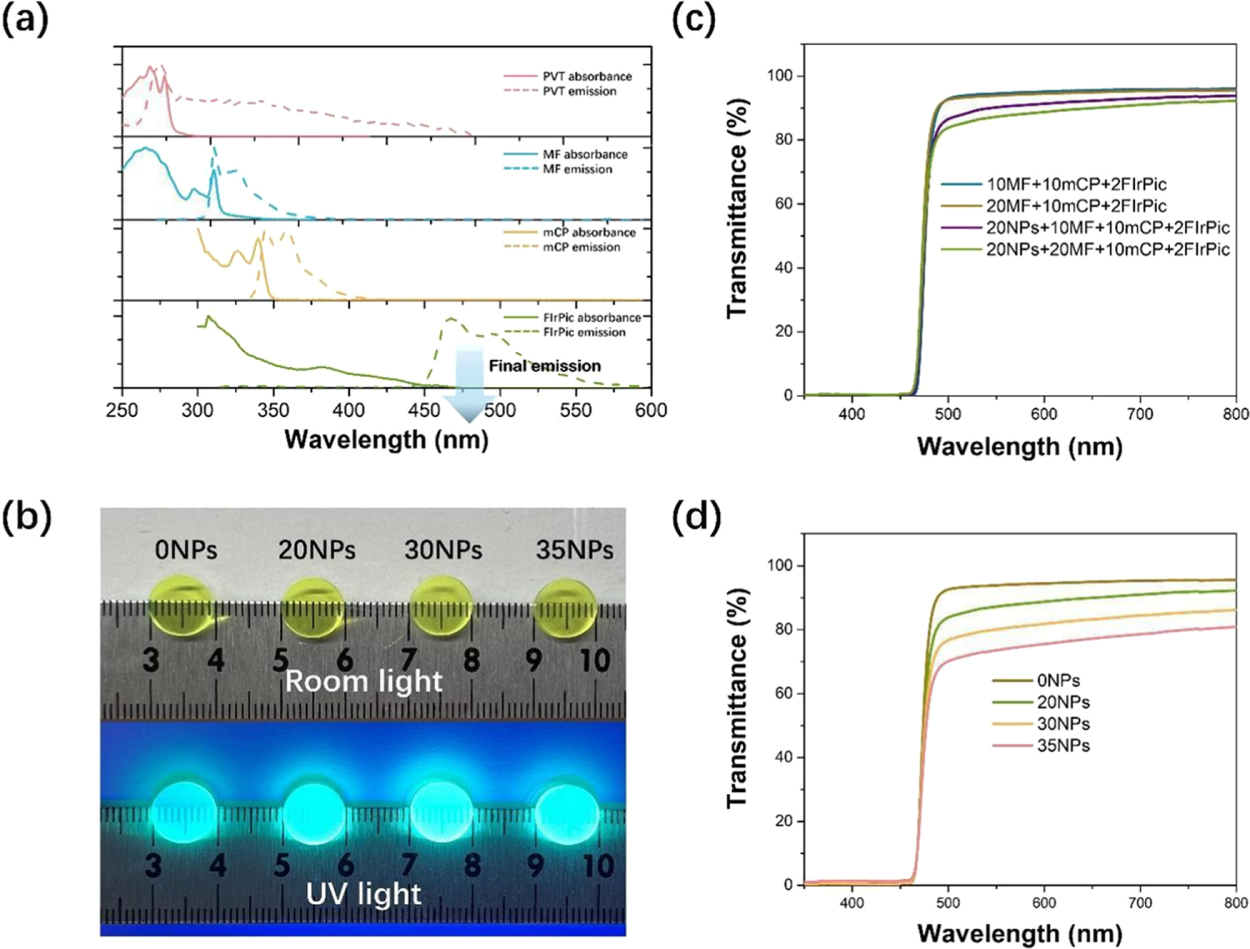
Optical properties of key components for plastic scintillators, optical images of samples, and transmittance curves of plastic scintillators and nanocomposites. (a) Absorbance (solid curve) and emission (dashed curve) spectra of PVT, MF, mCP, and FIrPic in cyclohexane. (b) From left to right: images of a plastic scintillator and nanocomposites (10 mm diameter, 2 mm thickness) containing 0–35 wt % nanoparticles (NPs), 20 wt % MF, 10 wt % mCP, and 2 wt % FIrPic under room light and under UV light. (c) Transmittance spectra of plastic scintillators and nanocomposites containing 0–20 wt % NPs, 10–20 wt % MF, 10 wt % mCP, and 2 wt % FIrPic. (d) Transmittance spectra of a plastic scintillator and nanocomposites containing 0–35 wt % NPs, 20 wt % MF, 10 wt % mCP, and 2 wt % FIrPic.

The absorbance and emission spectra of polyvinyltoluene (PVT), MF, mCP, and FIrPic in cyclohexane are shown in Figure 3a to examine the energy transfer between the key components within the plastic scintillators. FRET is effective over a distance of up to 10 nm, whereas Dexter energy transfer operates up to a distance of 1 nm.^17^ PVT has an emission maximum at approximately 270 nm, allowing efficient FRET to MF, which has an absorbance peak at approximately 260 nm. Therefore, FRET also occurs between PVT and mCP for the same reason. On the other hand, FIrPic is loaded at 2 wt %, which is sufficient for the phosphorescent molecules to be within 1 nm from MF, mCP, and PVT for efficient Dexter energy transfer if FIrPic dispersion in the matrix is uniform on the molecular level. As MF (∼3 eV) and mCP (2.9 eV) have slightly higher triplet energy levels than FIrPic (2.7 eV), both MF and mCP act as triplet hosts that can facilitate Dexter energy transfer of triplet excitons to FIrPic.^14,15,28^ From an energy transfer perspective, either MF or mCP alone would be a suitable host material to bridge the gap between PVT and FIrPic. Increased MF loading continues to enhance light yield when loaded up to 20 wt %, although any higher loading than this will lead to decreased light yield as a result of aggregation induced photoluminescence quantum yield (PLQY) quenching.^7^ Similarly, increased mCP loading appears to continuously enhance light yield, but it cannot be loaded at any value greater than 10 wt % due to its solubility limit in methylstyrene.

A photo of a plastic scintillator and nanocomposites with 0–35 wt % nanoparticles, 20 wt % MF, 10 wt % mCP, and 2 wt % FIrPic, under room light and under UV light, is shown in Figure 3b.

The transmittance spectra of plastic scintillators and nanocomposites containing 0–20 wt % nanoparticles, 10–20 wt % MF, 10 wt % mCP, and 2 wt % FIrPic are shown in Figure 3c, and the transmittance spectra of a plastic scintillator and nanocomposites containing 0–35 wt % nanoparticles, 20 wt % MF, 10 wt % mCP, and 2 wt % FIrPic are shown in Figure 3d. Transparency remains fairly high for all samples, even those loaded with 35 wt % nanoparticles, 20 wt % MF, 10 wt % mCP, and 2 wt % FIrPic. The use of both MF and mCP near their respective solubility limits allows us to maximize the total loading of the triplet hosts in the system. Overall, the sharp drop at approximately 480 nm is due to FIrPic absorbance, and the transmittance loss with increased MF loading or increased nanoparticle loading is due to scattering. As expected, greater transmittance loss occurs with increased nanoparticle loading.

### Gamma Light Yield and Gamma Response

Because of FIrPic’s long decay time of 1.2 μs,^29−31^ the rise time and flat top time were set to 2.2 and 3.2 μs, respectively, in contrast to previous methods typically reporting a rise time and flat top time of 1.0 and 0.5 μs, respectively.^7,8,26^ Additionally, acquisition live time was increased up to 9 h for certain samples to collect more counts, in contrast to previous methods typically reporting an acquisition live time of only 1 h.^7,8,26^

**Figure 4:**
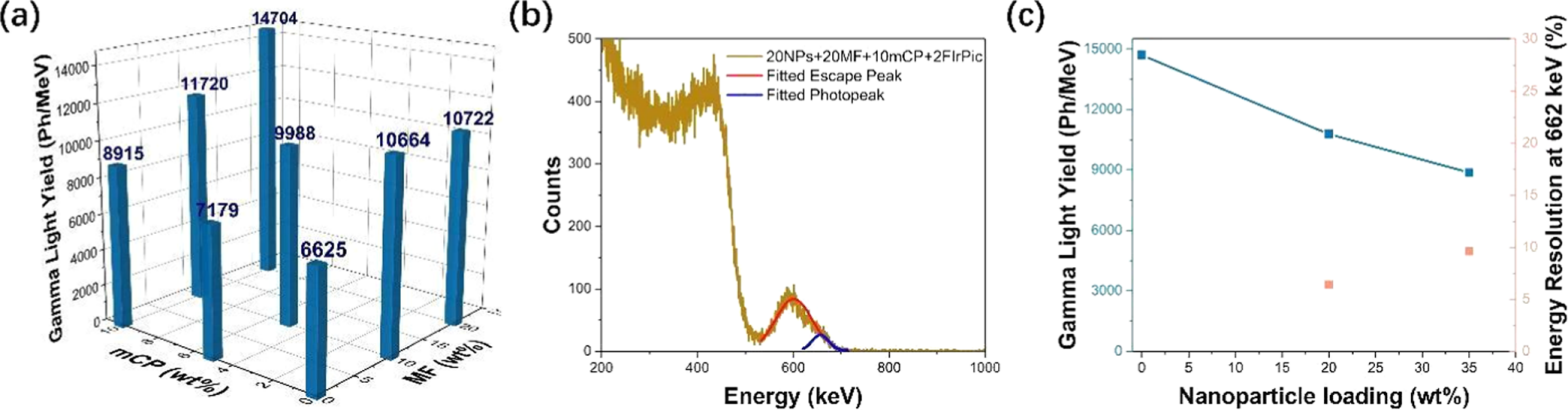
Gamma light yield and gamma response of plastic scintillators and nanocomposites. (a) Gamma light yield as a function of MF and mCP loading for plastic scintillators containing 2 wt % FIrPic. (b) Energy resolved pulse height spectrum of nanocomposite containing 20 wt % nanoparticles (NPs), 20 wt % MF, 10 wt % mCP, and 2 wt % FIrPic, with escape peak and photopeak deconvoluted. (c) Gamma light yield as a function of nanoparticle loading and energy resolution at 662 keV as a function of nanoparticle loading for nanocomposites containing 20 wt % MF, 10 wt % mCP, and 2 wt % FIrPic.

Plastic scintillators were prepared using 0–20 wt % MF, 0–10 wt % mCP, and 2 wt % FIrPic to investigate the optimized matrix formulation, and their gamma light yields are presented in Figure 4a. The sample containing 2 wt % FIrPic has the lowest gamma light yield of 6625 Ph/MeV. When loading either mCP or MF, the gamma light yield increases proportionally with the loading. Notably, the sample containing 10 wt % mCP and 2 wt % FIrPic has a gamma light yield of 8915 Ph/MeV, while the sample containing 10 wt % MF and 2 wt % FIrPic has a gamma light yield of 10 664 Ph/MeV. This difference is due to the more efficient FRET between PVT and MF, facilitated by the better overlapping of the emission spectrum of PVT and the absorbance spectrum of MF (Figure 3a). As 10 wt % mCP is the maximum possible loading due to its solubility limit in methylstyrene and MF cannot be loaded further than 20 wt % due to the previously mentioned aggregation induced PLQY quenching, the optimized matrix formulation contained 20 wt % MF, 10 wt % mCP, and 2 wt % FIrPic. This sample’s gamma light yield, approximately 14 700 Ph/MeV, surpasses that of current polyvinylcarbazole scintillators containing FIrPic, which have a gamma light yield just above 7000 Ph/MeV.^19−21^

The ^137^Cs gamma pulse height spectrum for a nanocomposite containing 20 wt % nanoparticles, 20 wt % MF, 10 wt % mCP, and 2 wt % FIrPic was acquired using an acquisition live time of 9 h. The gamma light yield is 10 780 Ph/MeV, the energy resolution of the hafnium Kα escape peak at 607 keV is 14.6%, and the energy resolution of the deconvoluted photopeak at 662 keV is 6.4%, as shown in Figure 4b. For a nanocomposite containing 35 wt % nanoparticles, 20 wt % MF, 10 wt % mCP, and 2 wt % FIrPic acquired with an acquisition live time of 5 h, the gamma light yield is 8863 Ph/MeV, the energy resolution of the hafnium Kα escape peak at 607 keV is 17.1%, and the energy resolution of the deconvoluted photopeak at 662 keV is 9.6% (Figure S2a). A plot of gamma light yield as a function of nanoparticle loading as well as energy resolution at 662 keV as a function of nanoparticle loading is shown in Figure 4c. As expected, the gamma light yield decreases with nanoparticle loading, but even in the nanocomposite containing 35 wt % nanoparticles, a gamma light yield of nearly 9000 Ph/MeV is retained. Energy resolution at 662 keV is less than 10% for both nanocomposites containing either 20 or 35 wt % nanoparticles.

To assess the scalability of the plastic scintillators and nanocomposites, samples with 17 mm diameter were prepared. A 17 mm diameter plastic scintillator containing 20 wt % MF, 10 wt % mCP, and 2 wt % FIrPic has a gamma light yield of 14 865 Ph/MeV, matching well with that of the 10 mm diameter sample with a gamma light yield of 14 704 Ph/MeV. Besides scaling up diameter, thickness was also increased in subsequent samples. A 17 mm diameter, 5.6 mm thick nanocomposite containing 20 wt % nanoparticles, 20 wt % MF, 10 wt % mCP, and 2 wt % FIrPic results in a gamma light yield of 9411 Ph/MeV, an energy resolution of the hafnium Kα escape peak of 19.3% at 607 keV, and an energy resolution of the deconvoluted photopeak of 9.7% at 662 keV (Figure S2c).

Overall, the modified organic matrix containing 20 wt % MF, 10 wt % mCP, and 2 wt % FIrPic has a gamma light yield of nearly 15 000 Ph/MeV. Even when loaded with 20–35 wt % nanoparticles, the nanocomposites still retain a gamma light yield of approximately 8800–10 800 Ph/MeV. This relatively high gamma light yield, both with and without nanoparticles, is possible due to the harvesting of triplet excitons. Typically, only some excitons are generated in the singlet state as predicted by random spin statistics, then converted into photons through fluorescent emission. In contrast, the remaining excitons are generated in the triplet state, but they are only able to transition from the excited state to the ground state in a nonradiative manner due to spin selection rules such that no photon is emitted.^10,11^ MF and mCP act as triplet hosts to facilitate Dexter energy transfer that effectively transfer triplet excitons to FIrPic, which is a phosphorescent dye. Therefore, both singlet excitons and triplet excitons may be utilized, and the gamma light yield is substantially enhanced.^12,13^

## Conclusions

Dissolving both MF and mCP in the PVT matrix maximizes the loading of the Dexter energy host for FIrPic. Plastic scintillators containing 20 wt % MF, 10 wt % mCP, and 2 wt % FIrPic have a gamma light yield of 14 865 Ph/MeV at 662 keV. Loading hafnium oxide nanoparticles lowers the gamma light yield of the resulting nanocomposites. However, at 20–35 wt % nanoparticle loadings, a gamma light yield between 8800 and 10 800 Ph/MeV is still retained, which is comparable to existing unloaded plastic scintillators. The loaded nanocomposites also show satisfactory energy resolution; nanocomposites with both 10 mm diameter and 17 mm diameter have a photopeak energy resolution less than 10% at 662 keV. Such performance is possible due to the introduction of MF and mCP as triplet hosts to facilitate Dexter energy transfer to FIrPic, a phosphorescent dye, such that both singlet excitons and triplet excitons are effectively utilized. Herein, phosphorescent emission becomes possible, which utilizes the previously lost triplet excitons and opens up the possibility to increase the gamma light yield compared to a system that only uses fluorescent emission.

## Supporting Information Available

^137^Cs gamma pulse height spectra; gamma light yield; energy resolution of plastic scintillators and nanocomposites; and calculation of Compton edge and energy resolution (PDF)

## References

[ref1] Dujardin C.; Auffray E.; Bourret-Courchesne E.; Dorenbos P.; Lecoq P.; Nikl M.; Vasil’ev A. N.; Yoshikawa A.; Zhu R. Y. Needs, Trends, and Advances in Inorganic Scintillators. IEEE Trans. Nucl. Sci. 2018, 65, 1977–1997. 10.1109/TNS.2018.2840160.

[ref2] Knoll G. F. Radiation Detection and Measurement; John Wiley & Sons, 2010.

[ref3] Siciliano E. R.; Ely J. H.; Kouzes R. T.; Milbrath B. D.; Schweppe J. E.; Stromswold D. C. Comparison of PVT and NaI(Tl) scintillators for vehicle portal monitor applications. Nucl. Instrum. Methods Phys. Res., Sect. A 2005, 550, 647–674. 10.1016/j.nima.2005.05.056.

[ref4] Koshimizu M. Recent progress of organic scintillators. Jpn. J. Appl. Phys. 2022, 62, 010503 10.35848/1347-4065/ac94fe.

[ref5] Hajagos T. J.; Liu C.; Cherepy N. J.; Pei Q. High-Z Sensitized Plastic Scintillators: A Review. Adv. Mater. 2018, 30, 1706956 10.1002/adma.201706956. 29736994

[ref6] Galib S. M.; Bhowmik P. K.; Avachat A. V.; Lee H. K. A comparative study of machine learning methods for automated identification of radioisotopes using NaI gamma-ray spectra. Nucl. Eng. Technol. 2021, 53 (12), 4072–4079. 10.1016/j.net.2021.06.020.

[ref7] Han Z.; Yu H.; Pei Q. Fluorene Derivatives for Efficient Prompt Scintillation in Plastic Scintillators. ACS Appl. Polym. Mater. 2022, 4, 4424–4431. 10.1021/acsapm.2c00391.

[ref8] Yu H.; Winardi I.; Han Z.; Prout D.; Chatziioannou A.; Pei Q. Fast Spectroscopic Gamma Scintillation Using Hafnium Oxide Nanoparticles-Plastic Nanocomposites. Chem. Mater. 2024, 36 (1), 533–540. 10.1021/acs.chemmater.3c02631.

[ref9] Feron K.; Belcher W. J.; Fell C. J.; Dastoor P. C. Organic Solar Cells: Understanding the Role of Förster Resonance Energy Transfer. Int. J. Mol. Sci. 2012, 13 (12), 17019–17047. 10.3390/ijms131217019. 23235328 PMC3546737

[ref10] Hu D.; Yao L.; Yang B.; Ma Y. Reverse intersystem crossing from upper triplet levels to excited singlet: a ‘hot excition’ path for organic light-emitting diodes. Philos. Trans. R. Soc., A 2015, 373, 20140318 10.1098/rsta.2014.0318. PMC4455719 25987570

[ref11] Pols M. C. W. M. Triplet-Triplet Annihilation in Organic Light Emitting Diodes: An Investigation of Phosphorescent Host-Guest Materials; Eindhoven University of Technology, 2018.

[ref12] Zhang Q.; Tsang D.; Kuwabara H.; Hatae Y.; Li B.; Takahashi T.; Lee S. Y.; Yasuda T.; Adachi C. Nearly 100% Internal Quantum Efficiency in Undoped Electroluminescent Devices Employing Pure Organic Emitters. Adv. Mater. 2015, 27, 2096–2100. 10.1002/adma.201405474. 25678335

[ref13] Chen J. X.; Tao W. W.; Chen W. C.; Xiao Y. F.; Wang K.; Cao C.; Yu J.; Li S.; Geng F. X.; Adachi C.; Lee C. S.; Zhang X. H. Red/Near-Infrared Thermally Activated Delayed Fluorescence OLEDs with Near 100% Internal Quantum Efficiency. Angew. Chem. 2019, 131, 14802–14807. 10.1002/ange.201906575. 31313424

[ref14] Mu H.; Jiang Y.; Xie H. Efficient blue phosphorescent organic light emitting diodes based on exciplex and ultrathin Firpic sandwiched layer. Org. Electron. 2019, 66, 195–205. 10.1016/j.orgel.2018.11.014.

[ref15] Swensen J. S.; Polikarpov E.; Ruden A. V.; Wang L.; Sapochak L. S.; Padmaperuma A. B. Improved Efficiency in Blue Phosphorescent Organic Light-Emitting Devices Using Host Materials of Lower Triplet Energy than the Phosphorescent Blue Emitter. Adv. Funct. Mater. 2011, 21, 3250–3258. 10.1002/adfm.201100586.

[ref16] Kim B. S.; Yook K. S.; Lee J. Y. Above 20% external quantum efficiency in novel hybrid white organic light-emitting diodes having green thermally activated delayed fluorescent emitter. Sci. Rep. 2014, 4, 6019 10.1038/srep06019. 25317855 PMC5377539

[ref17] Sudha K.; Sundharamurthi S.; Karthikaikumar S.; Abinaya K.; Kalimuthu P. Switching of Förster to Dexter Mechanism of Short-Range Energy Transfer in *meso*-Anthrylporphyrin. J. Phys. Chem. C 2017, 121, 5941–5948. 10.1021/acs.jpcc.6b13042.

[ref18] Kim J. W.; You S. I.; Kim N. H.; Yoon J. A.; Cheah K. W.; Zhu F. R.; Kim W. Y. Study of Sequential Dexter Energy Transfer in High Efficient Phosphorescent White Organic Light-Emitting Diodes with Single Emissive Layer. Sci. Rep. 2014, 4, 7009 10.1038/srep07009. 25388087 PMC4228348

[ref19] Campbell I. H.; Crone B. K. Efficient plastic scintillators utilizing phosphorescent dopants. Appl. Phys. Lett. 2007, 90, 012117 10.1063/1.2430683.

[ref20] Rupert B. L.; Cherepy N. J.; Sturm B. W.; Sanner R. D.; Dai Z.; Payne S. A. Bismuth-Loaded Polymer Scintillators for Gamma Ray Spectroscopy. MRS Online Proc. Libr. 2012, 137, mrss11-1341-u03-03 10.1557/opl.2011.1480.

[ref21] Cherepy N. J.; Martinez H. P.; Sanner R. D.; Beck P. R.; Drury O. B.; Swanberg E. L.; Payne S. A.; Hurlbut C. R.; Morris B. New Plastic Scintillators for Gamma Spectroscopy, Neutron Detection and Imaging. In 2017 IEEE Nuclear Science Symposium and Medical Imaging Conference (NSS/MIC) IEEE, 2017; pp 1–3.

[ref22] Liu C.; Hajagos T. J.; Kishpaugh D.; Jin Y.; Hu W.; Chen Q.; Pei Q. Synthesis of Transparent Nanocomposite Monoliths for Gamma Scintillation. In Hard X-Ray, Gamma-Ray, and Neutron Detector Physics XVII; SPIE: San Diego, California, United States, 2015; pp 93–101.

[ref23] Liu C.; Hajagos T. J.; Kishpaugh D.; Jin Y.; Hu W.; Chen Q.; Pei Q. Facile Single-Precursor Synthesis and Surface Modification of Hafnium Oxide Nanoparticles for Nanocomposite γ-Ray Scintillators. Adv. Funct. Mater. 2015, 25, 4607–4616. 10.1002/adfm.201501439.

[ref24] Jin Y.; Kishpaugh D.; Liu C.; Hajagos T. J.; Chen Q.; Li L.; Chen Y.; Pei Q. Partial ligand exchange as a critical approach to the synthesis of transparent ytterbium fluoride-polymer nanocomposite monoliths for gamma ray scintillation. J. Mater. Chem. C 2016, 4, 3654–3660. 10.1039/C6TC00447D.

[ref25] Chen Y.; Liu C.; Jin Y.; Hajagos T. J.; Kishpaugh D.; Zhuang Q.; Pei Q. Ytterbium Fluoride Loaded Plastic Scintillators for γ-ray Spectroscopy. In Hard X-Ray, Gamma-Ray, and Neutron Detector Physics XVIII SPIE, 2016; pp 82–93.

[ref26] Liu C.; Li Z.; Hajagos T. J.; Kishpaugh D.; Chen D. Y.; Pei Q. Transparent Ultra-High-Loading Quantum Dot/Polymer Nanocomposite Monolith for Gamma Scintillation. ACS Nano 2017, 11 (6), 6422–6430. 10.1021/acsnano.7b02923. 28551988

[ref27] Yu H.; Zhao R.; Redding C.; Chen T.; Hajagos T. J.; Ferrelli G.; Zaldivar R. J.; Hayward J. P.; Pei Q. Organic Liquid and Nanocomposite Scintillators for Gamma Spectroscopic Detections. In Hard X-Ray, Gamma-Ray, and Neutron Detector Physics XXIII; SPIE, 2021; pp 85–101.

[ref28] van Loef E. V.; Feng P.; Markosyan G.; Shirwadkar U.; Doty P.; Shah K. Triplet Harvesting Plastic Scintillators with Neutron-Gamma Pulse Shape Discrimination. In Hard X-Ray, Gamma-Ray, and Neutron Detector Physics XVI; SPIE: San Diego, California, United States, 2014 921306.

[ref29] Sanner R. D.; Cherepy N. J.; Young V. G. Blue Light Emission from Cyclometallated Iridium (III) Cyano Complexes: Syntheses, Crystal Structures, and Photophysical Properties. Inorg. Chim. Acta 2016, 440, 165–171. 10.1016/j.ica.2015.10.030.

[ref30] Woon K. L.; Hasan Z. A.; Ong B. K.; Ariffin A.; Griniene R.; Grigalevicius S.; Chen S. A. Triplet states and Energy Back Transfer of Carbazole Derivatives. RSC Adv. 2015, 5, 59960–59969. 10.1039/C5RA09340F.

[ref31] Kim J. H.; Yoon D. Y.; Kim J. W.; Kim J. J. New host materials with high triplet energy level for blue-emitting electrophosphorescent device. Synth. Met. 2007, 157, 743–750. 10.1016/j.synthmet.2007.08.001.

