# Supplementary material for: Nanocomposite Scintillators Loaded With Hafnium Oxide and Phosphorescent Host and Guest for Gamma Spectroscopy
Source: Chem Mater. 2024 May 7;36(10):5257–63. doi: 10.1021/acs.chemmater.4c00805 (PMC11137814; doi:10.1021/acs.chemmater.4c00805)
Supplement: Supplementary file 1 — cm4c00805_si_001.pdf [file cm4c00805_si_001.pdf]

Supporting Information

Nanocomposite Scintillators Loaded With Hafnium Oxide and Phosphorescent Host and Guest for Gamma Spectroscopy

*Isabelle Winardi,[‡] Ziqing Han,[‡] Hao Yu, Prabhav Surabhi, and Qibing Pei**

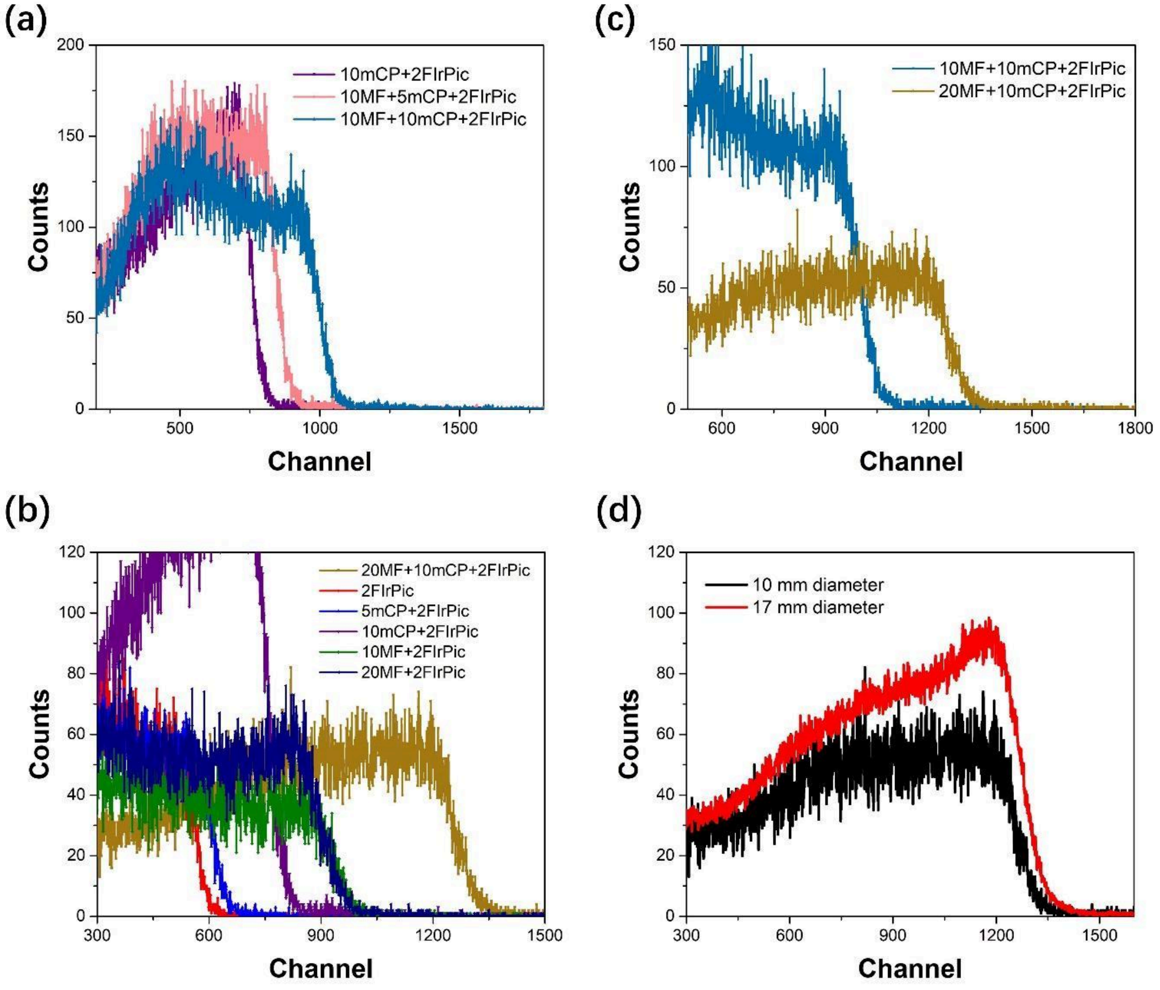

Figure S1. ^{137}Cs gamma pulse height spectra of plastic scintillators. (a) Pulse height spectra of plastic scintillators containing 0-10 wt% MF, 5-10 wt% mCP, and 2 wt% FIrPic. (b) Pulse height spectra of plastic scintillators containing 0-20 wt% MF, 0-10 wt% mCP, and 2 wt% FIrPic. (c) Pulse height spectra of plastic scintillators containing 10-20 wt% MF, 10 wt% mCP, and 2 wt% FIrPic. (d) Pulse height spectra of 10 mm diameter versus 17 mm diameter plastic scintillators containing 20 wt% MF, 10 wt% mCP, and 2 wt% FIrPic.

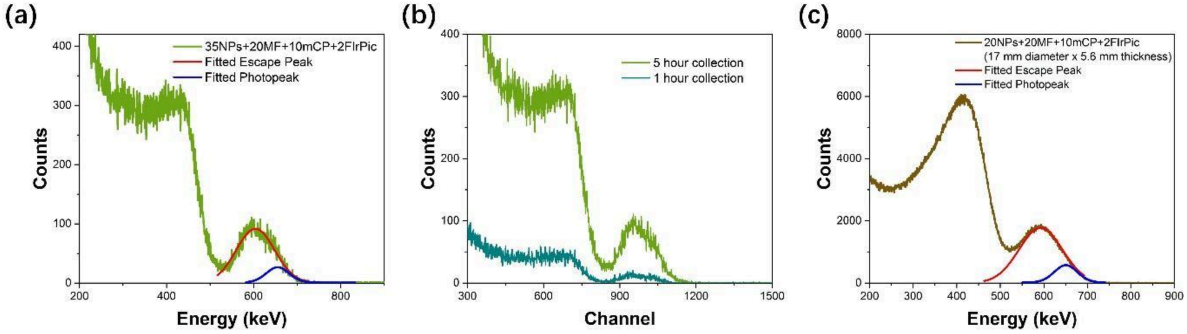

Figure S2. ^{137}Cs gamma pulse height spectra of nanocomposites. (a) Energy resolved pulse height spectrum of nanocomposite containing 35 wt% nanoparticles (NPs), 20 wt% MF, 10 wt% mCP, and 2 wt% FIrPic, with escape peak and photopeak deconvoluted. (b) Pulse height spectrum of nanocomposite containing 35 wt% NPs, 20 wt% MF, 10 wt% mCP, and 2 wt% FIrPic, comparing counts between 1 hour collection and 5 hour collection. (c) Energy resolved pulse height spectrum of 17 mm diameter, 5.6 mm thick nanocomposite containing 20 wt% NPs, 20 wt% MF, 10 wt% mCP, and 2 wt% FIrPic, with escape peak and photopeak deconvoluted.

Table S1. Gamma light yield and energy resolution of plastic scintillators and nanocomposites. All samples have 10 mm diameter and 2 mm thickness unless explicitly stated otherwise.

Sample	Gamma Light Yield (Ph/MeV)	Energy Resolution (Hafnium Kα escape peak at 607 keV)	Energy Resolution (Photopeak at 662 keV)
2% FIrPic	6,625	N/A	N/A
5% mCP, 2% FIrPic	7,179	N/A	N/A
10% mCP, 2% FIrPic	8,915	N/A	N/A
10% MF, 2% FIrPic	10,664	N/A	N/A
20% MF, 2% FIrPic	10,722	N/A	N/A
10% MF, 5% mCP, 2% FIrPic	9,988	N/A	N/A
10% MF, 10% mCP, 2% FIrPic	11,720	N/A	N/A
20% MF, 10% mCP, 2% FIrPic (10 mm diameter)	14,704	N/A	N/A
20% MF, 10% mCP, 2% FIrPic (17 mm diameter)	14,865	N/A	N/A
20% NPs, 20% MF, 10% mCP, 2% FIrPic (10 mm diameter)	10,780	14.6%	6.4%
20% NPs, 20% MF, 10% mCP, 2% FIrPic (17 mm diameter, 5.6 mm thickness)	9,411	19.3%	9.7%
35% NPs, 20% MF, 10% mCP, 2% FIrPic	8,863	17.1%	9.6%

Calculation of Compton Edge and Energy Resolution

A step by step sample calculation is shown for the 10 mm diameter, 2 mm thick nanocomposite containing 20 wt% NPs, 20 wt% MF, 10 wt% mCP, and 2 wt% FIrPic. All other nanocomposites followed the same calculation procedure. The plastic scintillators also followed the same calculation procedure as shown below except for the energy resolution calculations, which were not applicable for these samples.

The ^{137}Cs gamma pulse height spectrum was fitted to a curve using a smoothing function. The inflection point of the fitted curve was found by calculating the point at which its second derivative was equal to zero, and the x-axis value at the inflection point was taken as the Compton edge channel number. In the case of the 10 mm diameter, 2 mm thick nanocomposite containing 20 wt% NPs, 20 wt% MF, 10 wt% mCP, and 2 wt% FIrPic, whose pulse height spectrum is shown in **Figure S3**, the Compton edge channel number is 930.

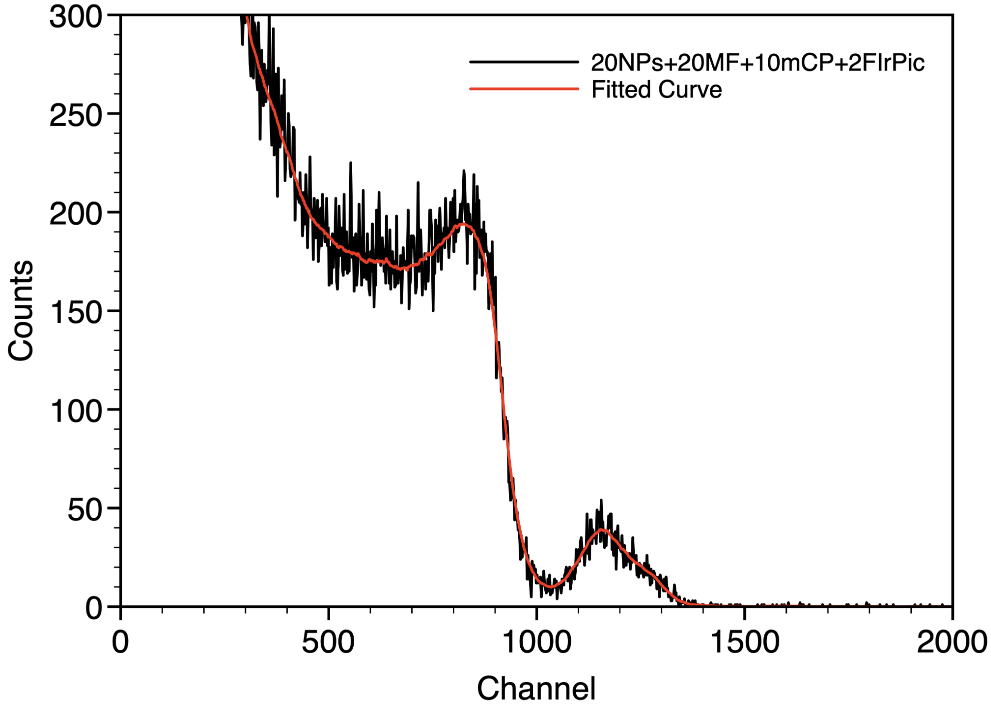

Figure S3. ^{137}Cs gamma pulse height spectrum of 10 mm diameter, 2 mm thick nanocomposite containing 20 wt% NPs, 20 wt% MF, 10 wt% mCP, and 2 wt% FIrPic.

For 662 keV γ rays from a Cs-137 source, the Compton edge corresponds to a deposited energy of 478 keV. Therefore, a correction factor α may be written as

$$\alpha = \frac{478\ keV}{Compton\ Edge\ channel\ number} \quad \textbf{(Equation S1)}$$

This multiplicative factor α may be used to correct the x-axis of a ^{137}Cs gamma pulse height spectrum plot. The corrected pulse height spectrum for the 10 mm diameter, 2 mm thick nanocomposite containing 20 wt% NPs, 20 wt% MF, 10 wt% mCP, and 2 wt% FIrPic is shown in **Figure S4**.

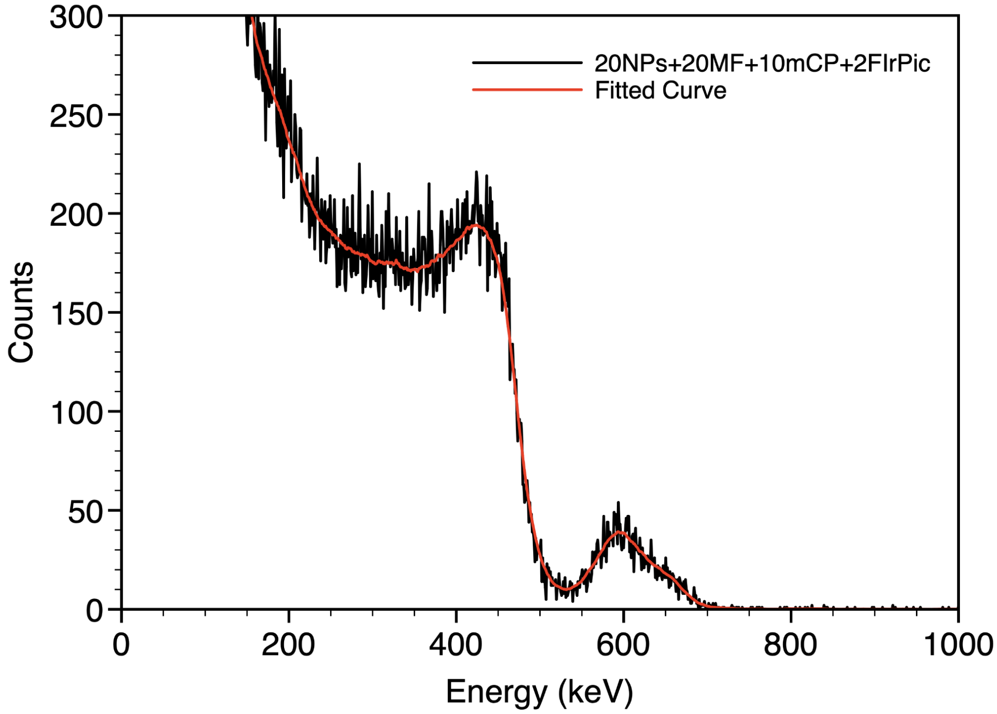

Figure S4. ^{137}Cs gamma pulse height spectrum of 10 mm diameter, 2 mm thick nanocomposite containing 20 wt% NPs, 20 wt% MF, 10 wt% mCP, and 2 wt% FIrPic, with corrected x-axis.

From here, the energy resolution of the hafnium Kα escape peak at 607 keV and the photopeak at 662 keV may be calculated. SciDAVis, a free application for **Sci**entific **D**ata **A**nalysis and **Vis**ualization, was used to perform the peak fittings. The multi-peak Gaussian fitting was used to

fit the hafnium Kα escape peak at 607 keV and the photopeak at 662 keV. For two peaks, the Gaussian equation may be written as

$$y = y0 + \sqrt{\frac{2}{\pi}} \bullet \frac{A1}{w1} \bullet e^{-2\bullet(x-c1)^2/w1^2} + \sqrt{\frac{2}{\pi}} \bullet \frac{A2}{w2} \bullet e^{-2\bullet(x-c2)^2/w2^2} \quad \textbf{(Equation S2)}$$

where *y0* is the offset, *A1* is the amplitude of the escape peak at 607 keV, *c1* is the center of the escape peak at 607 keV, *w1* is the width of the escape peak at 607 keV, *A2* is the amplitude of the photopeak at 662 keV, *c2* is the center of the photopeak at 662 keV, and *w2* is the width of the photopeak at 662 keV.

In the case of the 10 mm diameter, 2 mm thick nanocomposite containing 20 wt% NPs, 20 wt% MF, 10 wt% mCP, and 2 wt% FIrPic, the multi-peak Gaussian equation may be written as

$$y = 0.7 + \sqrt{\frac{2}{\pi}} \bullet \frac{3200.0}{82.1} \bullet e^{-2\bullet(x-596.4)^2/82.1^2} + \sqrt{\frac{2}{\pi}} \bullet \frac{294.4}{36.2} \bullet e^{-2\bullet(x-659.5)^2/36.2^2}$$

While the centers of the fitted peaks are not an exact match with the theoretical escape peak at 607 keV and photopeak at 662 keV, the percent errors are small (less than 2% error for the 607 keV escape peak and less than 0.5% error for the 662 keV photopeak).

The full width at half maximum (FWHM), necessary for the energy resolution calculation, is related to the Gaussian width as shown in **Equation S3** below.

$$\text{FWHM} = \frac{width\ (keV)}{0.849} \quad \textbf{(Equation S3)}$$

Then, the energy resolution (ER) is defined as shown in **Equation S4** below.

$$\text{ER} = \frac{FWHM\ (keV)}{662\ keV} \bullet 100\% \quad \textbf{(Equation S4)}$$

Using the experimental data, the ER may be calculated as follows.

$$\text{FWHM (607 keV peak)} = \frac{82.1\ keV}{0.849} = 96.7\ \text{keV}$$

$$\text{FWHM (662 keV peak)} = \frac{36.2\ keV}{0.849} = 42.6\ \text{keV}$$

$$\text{ER (607 keV peak)} = \frac{96.7\ keV}{662\ keV} \bullet 100\% = 14.6\%$$

$$\text{ER (662 keV peak)} = \frac{42.6\ keV}{662\ keV} \bullet 100\% = 6.4\%$$

The fitted peaks from SciDAVis may be superimposed onto the ^{137}Cs gamma pulse height spectrum with corrected x-axis, as shown in **Figure S5**.

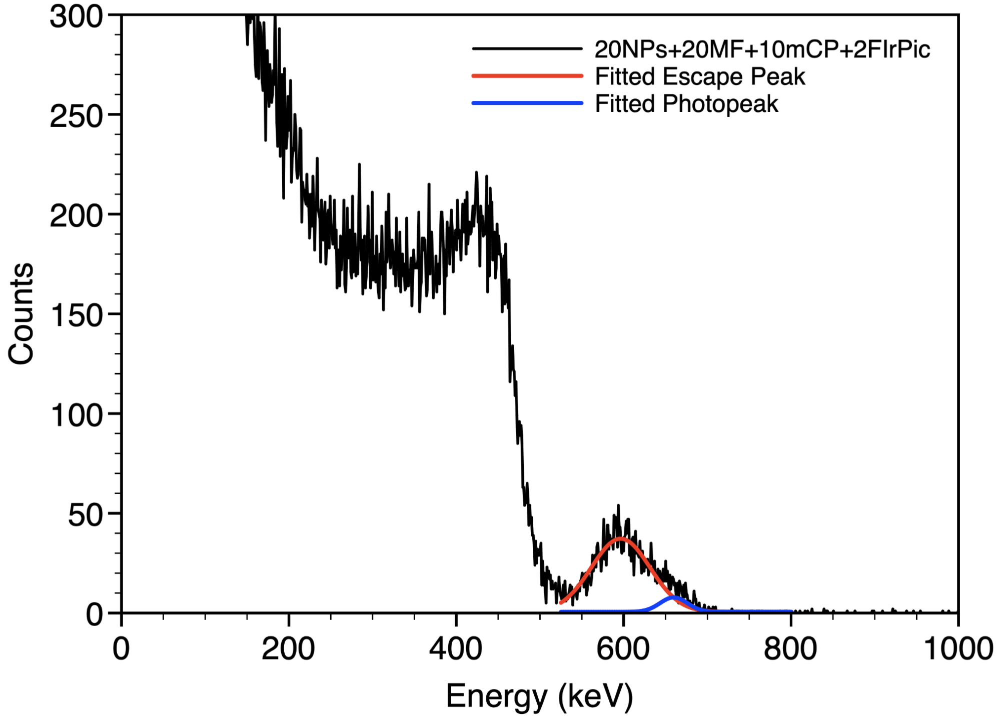

Figure S5. Energy resolved pulse height spectrum of 10 mm diameter, 2 mm thick nanocomposite containing 20 wt% NPs, 20 wt% MF, 10 wt% mCP, and 2 wt% FIrPic, with escape peak and photopeak deconvoluted.
